# S4MPLE—Sampler for Multiple Protein-Ligand Entities: Methodology and Rigid-Site Docking Benchmarking

**DOI:** 10.3390/molecules20058997

**Published:** 2015-05-19

**Authors:** Laurent Hoffer, Camelia Chira, Gilles Marcou, Alexandre Varnek, Dragos Horvath

**Affiliations:** 1 Laboratoire de Chemoinformatique; UMR 7141, Université de Strasbourg, 1 rue B. Pascal, Strasbourg 67000, France; E-Mails: hoffer.laurent@gmail.com (L.H.); g.marcou@unistra.fr (G.M.); varnek@unistra.fr (A.V.); 2 Novalix, BioParc, bld Sébastien Brant, BP 30170, Illkirch 67405 Cedex, France; 3 Department of Computer Science, Technical University, Cluj-Napoca 400027, Romania; E-Mail: camelia.chira@cs.utcluj.ro

**Keywords:** genetic algorithms, conformational sampling, docking, interaction fingerprints, force field fitting

## Abstract

This paper describes the development of the unified conformational sampling and docking tool called Sampler for Multiple Protein-Ligand Entities (S4MPLE). The main novelty in S4MPLE is the unified dealing with intra- and intermolecular degrees of freedom (DoF). While classically programs are either designed for folding or docking, S4MPLE transcends this artificial specialization. It supports folding, docking of a flexible ligand into a flexible site and simultaneous docking of several ligands. The trick behind it is the formal assimilation of inter-molecular to intra-molecular DoF associated to putative inter-molecular contact axes. This is implemented within the genetic operators powering a Lamarckian Genetic Algorithm (GA). Further novelty includes differentiable interaction fingerprints to control population diversity, and fitting a simple continuum solvent model and favorable contact bonus terms to the AMBER/GAFF force field. Novel applications—docking of fragment-like compounds, simultaneous docking of multiple ligands, including free crystallographic waters—were published elsewhere. This paper discusses: (a) methodology, (b) set-up of the force field energy functions and (c) their validation in classical redocking tests. More than 80% success in redocking was achieved (RMSD of top-ranked pose < 2.0 Å).

## 1. Introduction

Knowledge of the experimental structure of a target enables the use of structure-based drug design [1,2,3] (SBDD) methods. The key foundation of SBDD is computational prediction of geometries of interacting molecules, and their associated energy levels—in other words, conformational sampling (CS) [4,5,6]. This term covers everything from protein/peptide folding simulations to organic ligand conformer generation, to docking—albeit the latter is classically considered as a stand-alone chapter in SBDD. Docking [7,8,9,10] can be defined as the prediction of ligand-target complex structures at the atomic scale—CS of a super-molecular complex, in other words. There are three main docking simulations types, depending on considered degrees of freedom (DoF):

- rigid docking (target and ligand are considered rigid)
- semi-flexible docking (flexible ligand and rigid target)
- flexible docking (flexible ligand and partly flexible target)

Rigid docking programs are very fast but often inaccurate, since the required bioactive conformation of the ligand is rarely known. The program FRED [11] behaves as a fully rigid tool during the sampling step, but it uses a pool of conformers for each ligand. Most other tools explicitly sample ligand conformations during the docking process. Simultaneously, the search engine explores the different positions of the ligand within the rigid binding site, using translational and rotational degrees of freedom of the ligand. FlexX [12], Glide [13], DOCK [14] and Surflex [15] are widely used software of the semi-flexible category. Still, the hypothesis of a rigid target is often penalizing, since ligand binding often implies structural rearrangements of the target site, ranging from side chain to whole loop/subdomain displacements. There are different ways to include target flexibility. The most intuitive approach is to use an ensemble of different conformations of the receptor, and to perform semi-flexible docking on each target structure. Various conformations of the binding site obtained by X-ray or snapshots from molecular dynamics simulation can be used to create the collection of inputs. An alternative way is to generate a single united protein model, as in the FlexE strategy [16], from an ensemble of superimposed structures. In this model, the non-conserved regions are treated as alternative locations. Eventually, programs like Autodock [17,18] and Gold [19,20] may explicitly sample target DoF local flexibility, from rearranging polar hydrogens to sidechain rotations. Programs like RosettaLigand [21], FlipDock [22], FITTED [23,24,25] and IFD [26] allow for both backbone and sidechain flexibility. The real challenge remains to develop algorithms able to screen a database with flexibility in order to simulate conformational changes of the binding site (a phenomenon known as ‘induced fit’).

### 1.1. Sampling Strategies

In practice, a both exhaustive and time-effective exploration of the degrees of freedom of the whole receptor-ligand-solvent system is not possible. Molecular dynamics-based approaches [4,27,28] have a low propensity to bypass energy barriers, and are very slow to move the ligand into the constraining active site. However, they have a real physical basis, and are very useful during local optimization stages in order to find the closest local minima. Alternatively, popular stochastic heuristics such as Monte Carlo and evolutionary-based strategies are very general, so they can be adapted for many purposes, among them docking. Monte Carlo (MC) strategies [29] consist in several cycles of random modifications of a system coupled with a thermal bath, so that the acceptance probability of a random step is dictated by the Metropolis criterion (the Boltzmann term of the associated energy variation) at a given temperature.

Evolutionary/Genetic Algorithms (EA/GA) [10,30,31,32] rely on simulations of Darwinian evolution: ‘individuals’ or ‘chromosomes’ (vectors encoding points in the problem space to visit—here, coordinates of a molecule or of a molecular complex—are more likely to be selected into the next generation population if their fitness—here, the conformational stability—is higher. The chromosomes of the population undergo random modifications with biological operators such as mutation and cross-overs. Several docking tools, based on EA/GA, have been developed, among others GOLD, Autodock, FlipDock, EAdock [30,33], LGA [34] and FITTED. Since S4MPLE is GA-driven, the technical details thereof will be discussed in the Experimental section.

### 1.2. Scoring Functions

In-depth conformational sampling should, in principle, enumerate enough possible states to allow determination of the free energies of various conformers (understood as ensembles of geometries within a potential energy well). If so, then the free energy differences between bound and dissociated site-ligand states should directly lead to the binding affinity of that protein-ligand pair. In practice, this is not possible—both because sampling is never exhaustive enough, but mainly because the energy, function of the geometry, is insufficiently accurate. Therefore, most programs use scoring functions—basically QSAR equations trying to predict binding free energy as a function of a (typically, the lowest-energy) site-ligand pose. Such empirical scoring functions [8,35,36] are a weighted sum of ligand-receptor interaction terms. These often include hydrogen bonds, ionic bonds and hydrophobic contributions, clashes or even entropic penalties. Weights of each term of this QSAR equation are obtained by a regression analysis on a reference set of receptor-ligand complexes with known binding affinities, or from relative occurrence likelihoods in experimental structural databases. S4MPLE does so far not implement any free-energy scoring function: preferred poses are selected with respect to their force field energies only. This paragraph was inserted because S4MPLE will be compared to state-of-art docking problems which do use them.

### 1.3. Overview of This Article

S4MPLE has been conceived as a completely general conformational sampling program, specifically targeted at complex problems of the size of peptide folding and flexible docking problems. Its specificity is the generic approach to sampling, which transcends the traditionally accepted distinction made between folding and docking approaches. Full control over the considered and respectively frozen DoF allows S4MPLE to be used in either single-molecule sampling (up to small peptide folding), in any of the three main docking scenarios cited in the introduction, and beyond (protein loop repositioning in protein homology models, docking in presence of mobile protein loops, simultaneous or concurrent docking of several ligands into a same site, *etc.*). Previous publications concerning prior related developments were of technical nature, and considered the deployment strategies of the GA-driven conformational sampling algorithm on computer grids.

Specific difficult sampling and docking problems for which S4MPLE was originally designed were already published [37,38] or will be subject of independent publications. This report focuses on technical details and eventually outlines some of the classical benchmarking studies—a must when designing and validating a new sampling and docking tool. Addressed issues, in relative order of importance, are:

- The key novelty in S4MPLE: genetic operators supporting the unified handling of both intra- and inter-molecular DoF
- Differentiable interaction fingerprints to monitor conformer diversity—another important original development
- Employed evolutionary strategies, the originality of which draws on the novelty of involved operators and diversity control tools
- The set-up and fitting of the force field (FF) engine used for energy calculations, based on the classical AMBER [39,40]/Generalized AMBER (GAFF) [39,41]. The introduction of classical FF terms is combined with the introduction of—occasionally innovative, often adapted—additional terms: chirality control terms, a continuum solvent model and favorable contact (hydrophobic, H bonding) bonus terms. The latter, together with classical customizable FF terms—such as the chosen dielectric constant, and a herein introduced repulsive van der Waals coefficient weighing term—need fitting.
- Introduction of the herein used data sets.
- The fitting protocol of the additional terms. This may count as a conceptual novelty in as far as calibration efforts based on published ligand-protein complexes of known affinities were so far reserved to derivations of free energy-predicting scoring functions [7,42], not to fine-tune the FF engine *per se.*
- Eventually, the redocking protocol used to asses S4MPLE proficiency in classical docking benchmarks is given, in parallel to the similar protocol running FlexX, for comparison purposes.

## 2. Experimental Section

### 2.1. S4MPLE

S4MPLE is a flexible molecular modeling tool, based on a hybrid Genetic Algorithm (GA), combining molecular modeling-specific optimization and classical evolutionary sampling strategies. Allowing full control of the considered degrees of freedom, S4MPLE is a completely general approach to visit the conformational space of arbitrary molecules or molecular complexes. Its focus is on thorough geometry sampling, without need to rely on compound class-specific working hypotheses (amino acid rotamer libraries, *etc.*), potentially restraining its applicability domain. As such, it may be equally well used for conformational sampling and docking—which is nothing but sampling of a ligand in presence of a binding site. The site does however not need to be a protein, which makes S4MPLE useful for simulations of arbitrary molecule self-assembly processes. Being conceived in view of large-scale deployment on computer grids, the only limitations of its applicability are (a) the studied system size *vs.* available computational resources, and (b) the availability of force field parameters for the studied molecules. S4MPLE is written in object-Pascal, and used in command-line mode.

### 2.2. Hybrid Evolutionary Operators

S4MPLE represents the further development of an evolutionary sampling tool deployed on computer grids. This precursor [43] is a classical rigid rotamer approach, where the vector of torsional angle values assigned to each considered rotational axis formed the ‘chromosome’ of the problem (completed with Euler angles and translation vector values for docking). By contrast, herein considered full flexibility requires working with Cartesian coordinates of each atom. Or, the matrix *x_ij_*, with *i* being the atom index and *j = 1–3* the coordinate index (*j = 1* means ‘x’, *2* is ‘y’ and *3* is ‘z’) is a poor support for direct crossovers and mutations. Single-point mutations make little sense in this context (changing one coordinate of one atom at a time would lead to a locally distorted geometry of extremely high energy, yet similar to its parent). Plain crossing-over of two sets of Cartesian coordinates is bound to lead to physically impossible geometries, because, unlike torsional angle vectors, these are not invariant to roto-translation. The key challenge in the development of S4MPLE was the conception of genetic operators able to manipulate both intra- and inter-molecular DoF, in order to achieve the desired unification of sampling/folding and docking. The first step in this direction was, of course, abandoning the above-mentioned torsion-angle-centric approach in favor of full flexibility (all while maintaining a central role for torsional angles in the new strategy). These original cross-overs and mutation procedures will be introduced in the paragraphs following discussion of prerequisites related to atom flexibility status. Next, ‘Lamarckian’ local optimization operators (LO—classical gradient-based optimization-driven), are described. Eventually, this section concludes on a brief description of population initialization. 

#### 2.2.1. Atom Flexibility Status

By default, all atoms are considered flexible. Fixed atoms need to be explicitly listed in an input file. S4MPLE may input at most two distinct molecule files in mol2, car or sdf format, one of which may contain several species. When several molecules are input (for example, a protein site from mol2 and a set of competing ligands from sdf), the ‘site’ is assumed to be the species containing fixed atoms. S4MPLE starts by checking for rotatable (singe exocyclic) bonds, in order to break up the molecule into fragments, which constitute the ‘operands’ on which the generic operators will apply. By default, a minimal size of five atoms is required per fragment. Any single bonds (except amide, by default) exocyclic bond divides a molecule into two moieties. The smaller (amongst moieties not containing any fixed atoms) will preferentially become the fragment associated to that bond. When both moieties happen to contain fixed atoms, there will be no fragment associated to that bond. Therefore, some atoms may be not part of any fragment, without being fixed (their geometry may change during gradient optimization, for example). Let us refer to these as ‘passive’ moieties. 

A ring system will typically count as a single fragment, and intra-cyclic bonds are not considered as recombination points either. This behavior can be changed by formally declaring one of the ring bonds as ‘broken’. Using this trick, S4MPLE will specifically ignore a given bond during the fragment list build-up, but its harmonic FF contribution is not modified. The consequence is the ability to sample ring conformations. Likewise, if a bond within the path connecting two fixed moieties (example: a peptide bond within a loop connecting two fixed helices) is ‘broken’, atoms in that loop lose their passive status, and are incorporated in regular fragments.

Disjoined molecular graphs void of any fixed atoms are considered as stand-alone molecules (e.g., ligands). They will be rendered as non-covalently connected fragments, assuming one of the putative favorable inter-fragment contacts to play the role of connector instead of the covalent bond—see further on.

#### 2.2.2. Cross-Over Operators

In the following, molecule crossover, producing a chromosome child: = **CROSS** **(**parent_1_, parent_2_**)**, shall refer to a generic procedure taking two mating individuals as arguments, and randomly calling one of the two recombination operators designed here: *fragment recombination*, preferentially used in 80% of cases, and the alternative *uniform torsional crossover*. Eventually, it performs a LO (gradient relaxation).

**Figure 1:**
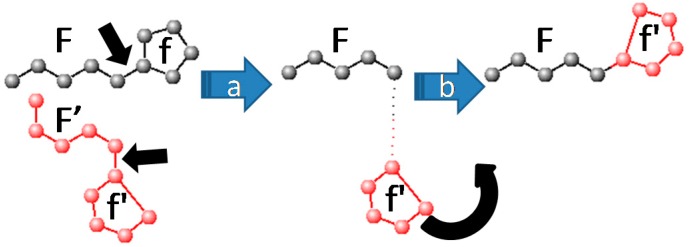
Principle of fragment recombination.

- *Fragment recombination* randomly picks a pair of complementary molecular fragments (**F**, **f**) defined at the beginning of the simulation. Such fragments may correspond either to two radicals (**F** containing more atoms than **f**), covalently connected to each other by a rotatable bond (see Figure 1), or a loose ligand **f** moving freely with respect to the receptor moiety **F**. In this way, folding and docking, the two key applications typically dealt with by different software suites, are here unified. The fragment recombination takes place in several stages. First (step a in Figure 1) the geometry of **F** (*x_ij_*, atom *i*∈**F**, coordinate *j = 1–3*) is taken from the first mate, whereas the geometry of **f** is imported from the second, as **f'**. Next (step b), **f'** will be reconnected to **F**, *i.e.* the imported coordinates (*x_ij_, i*∈**f'**) are submitted to a roto-translation meant to replace them in a chemically meaningful way with respect to **F**.

For bound fragments this means restoring valence bond length and angles around the cut bond to chemically acceptable values. For loose ones, the role of the chemical bond to be restored is formally assigned to a randomly picked hydrophobic contact or hydrogen bond involving one atom from **F** and another from **f'**. 

By default, S4MPLE would randomly try to realize any of the putative favorable contacts between them. Practically, however, when **F** is a large biomolecule, it will offer very many putative contact partners. Unbiased browsing through all these possibilities to suggest possible ligand placements (all around the protein surface)—would however represent a huge waste of time. Therefore, a **hot_spots** file enumerating the eligible contact partners of the active site (preferentially at the cleft bottom) is used to proactively orient the ligand towards the binding cleft. On the ligand side, all carbons, acceptors and donors count as putative contacts. First, a putative pair of matching contact atoms **a**∈**F** and **a'**∈**f'** (hydrophobe-hydrophobe or acceptor-donor) is selected. Next, for both **a** and **a'** S4MPLE tries to find a random solvent-accessible point on each of the contact spheres surrounding these atoms at vdW plus solvent probe radius (here 1.1 Å, slightly less than the water probe radius of 1.4 Å: slight clashes may be tolerated in initial poses). If detection of such an accessible point fails, for either **a** or **a'**, then the pair (**a**, **a'**) is dropped and the algorithm then tries to pick another—until all the 50 pair picking trials are exhausted. Suppose the selected accessible points were **P** and **p'** respectively. Partners are then brought within contact distance, *i.e.* the atom **a'** is placed at **P**. Next, **f'** is rotated in order to render vectors **a'**–**p'** and **a**–**P** antiparallel—meaning that **f'** is being tentatively kept, as much as possible, outside of the exclusion volume of **F**.

The above-mentioned constraints for rearrangement of **f'**, for both fragment junction types (covalent and loose), do not completely define the new geometry of the child structure. In particular, **f'** is free to rotate around the newly formed **F**–**f'** bond (or contact axis). This torsional degree of freedom may be determined according to two alternative fragment fitting procedures:

○ *DockC* randomly rotates **f'** around the **F**–**f'** axis, either until a clash-free arrangement is obtained, or a maximal number of attempts *(50)* is reached.
○ *DockE* pursues random rotations while attempting to minimize the inter-fragment interaction energies. It stops and returns the most stable pose found so far if *20* successive attempts failed to discover any better one.

*DockE*, the more resource-consuming of the two approaches, is randomly called in 30% of cases. Eventually, the geometry returned by *DockC/DockE* is submitted to LO. Note that LO may dramatically modify geometry, and therefore the genetic material of parents is not necessarily preserved in the child.

- *Uniform torsional crossovers* browse through the list of rotatable bonds, randomly pick one of the two parents as “donor” and set the associated torsion angle value in the child geometry equal to the one of the donor. This is a usual type of crossing-over operator. LO is then used to tentatively relax the atom clashes in the brute geometry associated to the combined list of torsion angle values.

#### 2.2.3. Mutations

*The Mutation* operator child: =**MUT** (parent) first randomly picks a pair of fragments (**F**, **f**), then checks whether these are bound or loose fragments. In the first case, a torsional angle associated to the inter-fragment bond is forced to change, either randomly or by means of a temporary constraint term in the energy function, followed by gradient optimization of geometry on this perturbed energy surface (“driven” mutations as reported previously [43]). Otherwise (loose fragment case), mutation is performed as a crossover of the molecule with itself. In other words, there is a repositioning of **f** with respect to **F**, allowing new inter-fragment contacts to be established, and leading to novel geometries.

#### 2.2.4. ‘Lamarckian’ Local Optimization

Occasional gradient-based local optimization (a.k.a ‘Lamarckian’ local optimization), during the evolutionary sampling procedure, is mandatory for molecules. This is due to the extreme ruggedness of the energy function. It is unwise to wait for a very long time until an appropriate mutation alleviates the bad contact, when few steps of gradient-based optimization may instantly solve the problem. All genetic operators will include, as a last step, a random number (between 20 and 50) of conjugate gradient (CG) relaxation iterations. These procedures will be generically referred to as LO (Local-or Lamarckian-Optimization).

#### 2.2.5. Exhaustive Energy Minimization

This operator is a succession of various descent methods (SD, CG, BFGS), alternatively applied to (a) the actual energy and (b) to a modified energy landscape, with the weight of the bond stretching contributions downscaled from the default 1.0 to some random value within (0.0, 0.2). Softened bonds allow for a temporarily less rugged potential, allowing gradient-based methods to escape irrelevant local minima. Successive minimization cycles are performed, toggling nominal and softened bond terms, until convergence. This approach is employed to refine so-far best sampled geometries before output, or at post-processing stages.

#### 2.2.6. Initialization

*Random Initialization* (RI) consists in scrambling the current geometry of the molecule object in memory by random rotations around torsional axes. Next, each fragment is tentatively rearranged in a clash-free manner (*DockC*). LO completes the procedure.

### 2.3. Population Diversity Control: Interaction Fingerprints

Any population-based heuristics is strongly tributary to a population diversity control mechanism. In absence of such, the risk of premature convergence is very important (via accumulation of minor mutants of a dominant, local energy minimum). S4MPLE adopts the postulate that two geometries may be considered redundant if they share a same set of contacts. This postulate is embodied by novel, fuzzy and differentiable Pairwise Interaction Fingerprints (PIF). Unlike classical ligand-protein IF used in docking [44,45,46], PIFs are:

- general, regrouping both intra- and intermolecular favorable contacts: hydrogen bonds and hydrophobic contacts
- symmetry-compliant, *i.e.* invariant to swapping of the contact status of topologically equivalent atoms: rotation of 180° of a carboxylate group having one of its oxygens acting as acceptor in a hydrogen bond will not change the fingerprint, as the hydrogen bond now involves a different, yet topologically equivalent oxygen.
- differentiable, rather than binary: contact status varies smoothly between 0 (absent) and 1 (fully established) as the corresponding contact distance scans [d_min_, d_max_].

Building this fingerprint, for a given molecular geometry, relies on preliminary atom typing work (done at the molecule input stage). This includes, first, detection of hydrophobes (carbons), hydrogen bond donors and acceptors. Assignment into the latter two categories is based on the AMBER/GAFF force field types:

- H-bond donors : H, HO, HW, hn, ho, hw
- H-bond acceptors : O, O2, OW, OH, OS, NB, NC, NY, o, oh, os, ow, n1, n2, n3, na, nb, nc, nd, ne, nf

Atoms of types others than listed above or amongst the considered classes of hydrophobes (detailed in Table 1) are not accounted for in PIFs. Next, ‘symmetry sets’ *S_k_* of topologically equivalent atoms are built—let their number be *k = 1...N_S_*_,_ while *N* denotes the total number of atoms. Atoms are topologically equivalent if they are of the same element, same partial charge *Q_i_* and have identical values of their below-given topological indices. These (*I_i_^1^, I_i_^2^*) capture the environment of the atom *i* in the molecular graph (topological distances, *i.e.* the shortest path-based number of bonds between, *i* and *j*, are labeled *T_ij_*). For generality, atoms from disjoined moieties (*i.e.* i in ligand, j in the protein) are set to infinity (practically, *T_ij_ = 999*).

**Table 1:** Description of the AMBER/GAFF carbon types and their classes.

Category	Force Field	Atomic Type	Description (from AMBER/GAFF Parameters File)
Polarized	AMBER	C	sp2 C carbonyl group
	GAFF	c	sp2 C carbonyl group
Aromatic	AMBER	C*	sp2 arom. 5 memb.ring w/1 subst. (TRP)
	AMBER	CA	sp2 C pure aromatic (benzene)
	AMBER	CB	sp2 aromatic C, 5&6 membered ring junction
	AMBER	CC	sp2 aromatic C, 5 memb. ring HIS
	AMBER	CN	sp2 C aromatic 5&6 memb.ring junct.(TRP)
	AMBER	CR	sp2 arom as CQ but in HIS
	AMBER	CV	sp2 arom. 5 memb.ring w/1 N and 1 H (HIS)
	AMBER	CW	sp2 arom. 5 memb.ring w/1 N-H and 1 H (HIS)
	GAFF	ca	sp2 C in pure aromatic systems
	GAFF	cc	sp2 carbons in non-pure aromatic systems
	GAFF	cd	sp2 carbons in non-pure aromatic systems
	GAFF	cp	Head sp2 carbons connecting rings in bi-phenyls
	GAFF	cq	Head sp2 carbons connecting rings in bi-phenyls
Aliphatic	AMBER	CT	sp3 aliphatic C
	GAFF	c1	sp C
	GAFF	c2	sp2 C
	GAFF	c3	sp3 C
	GAFF	ce	Inner sp2 carbons in conjugated systems
	GAFF	cf	Inner sp2 carbons in conjugated systems
	GAFF	cg	Inner sp carbons in conjugated systems
	GAFF	ch	Inner sp carbons in conjugated systems
	GAFF	cu	sp2 carbons in triangle systems
	GAFF	cv	sp2 carbons in square systems
	GAFF	cx	sp3 carbons in triangle systems
	GAFF	cy	sp3 carbons in square systems

**Figure 2:**
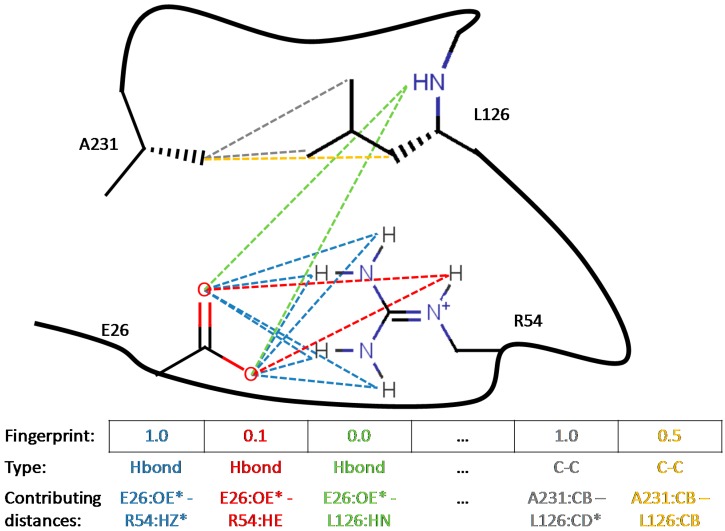
Principle of Interaction Fingerprints: each element represents a unique putatively favorable interaction, which may be embodied by different topologically equivalent atoms. For example, the E26-R54 ionic contact above is ‘on’ (associated PIF element set to 1.0) as soon as either of equivalent HZ/R54 are within contact distance of either of OE/E26 (* meaning ‘either atom’). For all distances between equivalent atoms (dotted lines of a same color) the corresponding contact strength are calculated and eventually averaged according to Equation (7). This biased average (favoring strong contact contributions) is reported in the associated PIF cell (color matching distance lines).

Note that these symmetry classes are also employed by S4MPLE to calculate symmetry-compliant root-mean-square-deviation (RMSD) values of atomic coordinates (not further detailed here—additional information available upon request). See the Supplementary Material for the pseudo-code describing the symmetry set build-up. Figure 2 exemplifies the mapping of monitored contacts in the fingerprint.

Note that any member *i* of a set *S* is equally distant, in terms of topological distance, from any other *j*∈*s*. Therefore, the topological distance *T* between symmetry sets can be defined as:

(1) $$T\left(S,s\right)\;=\;T_{ij}\;\;\;\forall \;i\;\in \;S\;and\;\forall \;j\;\in \;s$$

The contact fingerprint is then built with respect to all pairs of sets (*s*, *S*) where either both *s* and *S* are sets of hydrophobic carbons, or *s* are acceptors while *S* are donors. Only pairs of sets with at least one free atom, and not topologically too close (*i.e.* always close in space), are selected: *T(s, S) > 5*. Therefore, the fingerprint dimension *N_F_* equals to the total number of set pairs above. The PIF is thus defined as a vector of *k = 1 – N_F_* real values, in which every element *PIF_k_* monitors the contact strength associated to a pair *(s_k_, S_k_)* of symmetry sets fulfilling the above condition.

Element *PIF_k_* is thus a function of coordinates of a variable number of atoms *i∈S_k_* and *j∈s_k_*. Pairwise contact intensity *C_ij_* for each atom pair *(i, j)* can be inferred from Equation (5) below. Then, *PIF_k_* could be chosen to equal the *maximal* of all possible pairwise *C_ij_*. Unfortunately, this would not be a differentiable function. Therefore—except for the trivial case when *PIF_k_ = 0* because all *C_ij_ = 0*—the self-weighed average of contacts is used to define *PIF*:

(2) $$PIF_{k}=\frac{\sum_{i\in S_{k}}\sum_{j\in s_{k}}C_{ij}^{2}}{\sum_{i\in S_{k}}\sum_{j\in s_{k}}C_{ij}}$$

The main application of PIFs is the geometry redundancy check. Used by the default evolutionary strategies, this basically amounts to calculating the fingerprint size-relative block distance $\delta \left(g,G\right)=\sum_{k=1}^{N_{F}}\left|PIF_{k}^{G}\;-\;PIF_{k}^{g}\right|/N_{F}$ between two geometries *g* and *G*, each encoded by a chromosome in the population. This block distance represents a generic fraction of contacts having different status in *G* and *g* respectively. A user-defined *minfpdiff* cutoff (typically 0.01 for rigid site docking) is used to define geometry redundancy: *g* and *G* are redundant if the status of more than 99% of the theoretically possible contacts monitored in the PIF is unchanged.

The degree of originality *ω(G)* of an individual *G* is defined as the smallest δ(g,G) between *G* and any other *fitter* individual *g*. In other words, if *G* and *g* encode near identical geometries, but geometry g is energetically more stable, then *G* alone will be assigned a low originality score and replaced by the diversification strategy. Two distinct routines called **ReplaceMostRedundant** and **RandomizeIfRedundant** are in charge of population diversification, controlled by *minfpdiff*.

**ReplaceMostRedundant** allows an offspring *O* with a high degree of originality to potentially replace one of the redundant individuals in the population. The eligibility of *O* as a potential replacement increases with its originality: if *ω(O)*>*minfpdiff,* the child *O* is certain to participate, rather than strictly compete against its parents. The most redundant member (*MR*) of the current population is determined as follows:

- first, *ω_min_ = min[ω(M)]*—the minimal degree of originality over all members *M* of the population—is found. *MR* is taken to be the least fit individual among the least original members M (those with *ω(M) ≤ 1.1ω_min_*).
- An empirical trade-off between diversification and acceptation of less fit individuals is achieved: individual *O* replaces *MR* if its excess energy *E(O)−E(MR)* in kcal/mol is ‘compensated’ by the gain in diversity, at user-defined *div2E* parameter. If *E(O)−E(MR) ≤ div2E[ω(O)−ω(MR)]* and *ω(O) > ω(MR),* then the individual *O* replaces *MR*, and an offspring is accepted in the population and coexist with its parents. By default, children only replace their parents.

**RandomizeIfRedundant** can be applied to each member of the population, once per generation, after mutations, crossovers and replacements. Depending on the degree of originality *ω(I)* of the current individual *I*, of energy *E(I)*, this will be considered for randomization if *rand() < ω(I)/minfpdiff*. Attempts to randomly generate a new individual *M* of energy *E(M)* and originality are made, until

- *M* represents an acceptable trade-off between fitness loss *vs.* originality gain with respect to *I*: *E(M) − E(I) < div2E[ω(M) − ω(I)],* or
- the allotted number of attempts (5) is reached.

### 2.4. Evolutionary Strategies

Several evolutionary strategies were built on the basis of the above operators—three of which are described here. They are based on a set of standard procedure calls (described above as genetic operators). The pseudo code from Appendix 2 depicts a standard genetic algorithm (SGA), while the code from Appendix 3 depicts a simple evolutionary procedure (‘evol’). The term **MightReplace** stands for the standard challenge of the parents by the offspring. If an offspring is not original enough to enter the population by replacing a redundant individual (see **ReplaceMostRedundant**), it may challenge its parents on basis of its fitness score alone: if fitter, it will replace its least fit parent.

Alternatively, an elitist strategy ensures the survival of the fittest individuals in the next generation. The considered size of the elite subpopulation is 20% of the entire population. At each generation, the population is ranked based on the energy function and the best fitted chromosomes are declared elite, and cannot be modified or replaced. The general scheme of the considered Elitist Genetic Algorithm (*EGA*) is the same with that of SGA with the main difference that an elite individual will not be replaced by any of the *ReplaceMostRedundant* or *RandomizeIfRedundant* procedures. 

### 2.5. Force Field

Two force fields are currently implemented in S4MPLE:

- CVFF (Consistent Valence Force Field) [47], which has been imported from the previous work on a torsion-driven sampling tool [43,48] and,
- AMBER/GAFF, exclusively used in the herein reported results. AMBER [40] is widely used to simulate proteins and nucleic acids. Recently, its authors published GAFF, an extension for arbitrary organic molecules [41], comparable in accuracy to other force fields such as MMFF94 [49] or Tripos FF [50].

#### 2.5.1. Continuum Solvent Model and Contact Terms

In addition to the classical *in vacuo* force field terms, S4MPLE includes a specific continuum solvation model of maximal simplicity, in order to minimize its computational cost. It relies on simple functions of inter-atomic distances:

- A pair-based desolvation term (Equation (3)) [51], function of partial charges *Q* and distance *d* between two atoms *i* and *j*, and scaled by a generic constant (more about this in the force field fitting paragraph):
  (3) $$Edesolv_{\;ij}=\;\sigma \frac{{Q_{i}}^{2}+{Q_{j}}^{2}}{d_{ij}^{4}}$$
- A linearly distance-dependent relative dielectric constant (*ε_r_*) is used in the Coulomb term [52], which *de facto* makes it a function of *1/d^2^*:
  (4) $$ECoulomb_{\;ij}=\frac{332\;Q_{i}Q_{j}}{\varepsilon_{r}d_{ij}}$$
- Contact terms (Equation (5)) [51], rewarding favorable interactions such as hydrophobic contacts and hydrogen bonds were added to the FF. In this context, hydrophobic contacts refer to close carbons in space, and hydrogen bond donors are hydrogens on heteroatoms. Constant *κ_ij_* is a function of the nature of the contact, and the types of involved atoms *i* and *j*, see parameter fitting below. *C_ij_* encodes contact strength: full contact *C_ij_ = 1* is assumed at *d_ij_ < d_min_*. Contact ceases completely at *d_ij_ > d_max_*, and its strength varies smoothly within the switching range [d_min_, d_max_]. This range is contact type dependent: for hydrophobic contacts, a range of [4.5, 5.5] Ǻ is used. For hydrogen bonds, the range is atom pair specific: [*(sum of vdW Radii) − 0.5, (sum of vdW Radii) + 0.1*]:
  (5) $$Econtact_{ij}=\kappa_{ij}C_{ij}=\kappa_{ij}\left[0.5+0.5*cos\left(\pi \frac{d_{ij}^{2}-d_{min}^{2}}{d_{max}^{2}-\;d_{min}^{2}}\;\right)\right]$$

Since these terms are not included into the native FF, the additional parameters needed calibration, as outlined in the dedicated chapter below.

#### 2.5.2. Context-Specific Termination Function

S4MPLE uses pair-specific cut-off values: *cut_ij_* is determined by backwards scanning the distance range, starting from *maxcut = 15* Å, towards ever shorter distances, until the calculated pairwise energy contribution, all terms confounded, exceeds *minPairContrib = 0.01 kcal∙mol^−1^* This typically happens within the 10–12 Å range for pairs featuring strong Coulomb contribution, down to ~6 Å for vdW-dominated interactions. In order to avoid cut-off artifacts, a termination function $\omega_{ij}=1-2d_{ij}^{2}/cut_{ij}^{2}+d_{ij}^{4}/cut_{ij}^{4}$ is employed. Total non-bonded contributions can be written as:

(6) $$Enonbonded_{ij}=\omega_{ij}\left(ECoulomb_{ij}+EvdW_{ij}+Edesolv_{ij}\;+\;Econtact_{ij}\right)$$

#### 2.5.3. Out-of-Plane and Chiral Constraint Terms

**Figure 3:**
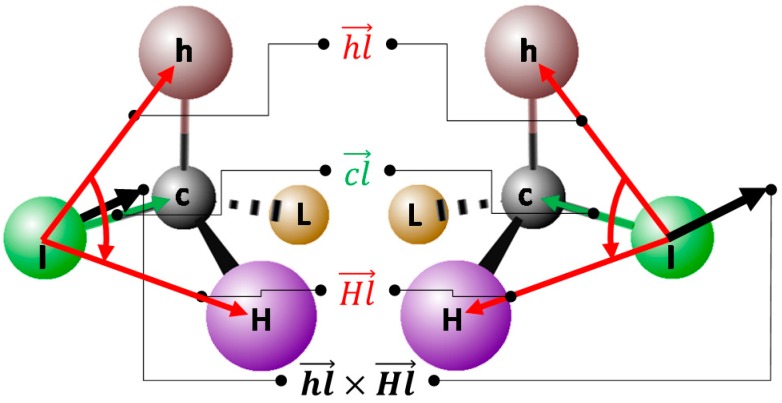
Calculation of the chirality index used to preserve the configuration of asymmetric carbons.

Random jumps in problem space may lead to distorted geometries, which may relax by chiral center inversion. Chiral constraints are defined with respect to the initial configuration of chiral carbons, and are strictly equal to zero unless the chiral carbon geometry approaches the planar state required for inversion. Because this is a relative term aimed to preserve the initial configuration, the priorities of the four substituents (***L***owest, ***l***ow, ***h***igh, ***H***ighest) of the chiral center may be arbitrary—they are taken according to the internal atom numbering (see Figure 3). 

An arbitrary face of the tetrahedron (say *l, h, H*) is selected, and a chiral direction vector, orthogonal to the *(l, h, H)* plane is defined as $\vec{Hl}\times \vec{hl}$. Here, the vectors represent relative position vectors of corners *H* and *h* with respect to *l*. Eventually, the position vector of the chiral center c with respect to *l*, $\vec{cl}$, is computed, and projection of $\vec{cl}$ along the direction $\vec{Hl}\times \vec{hl}$ is estimated as the dot product of these normed vectors:

(7) $$=\vec{cl}∙\left(\vec{Hl}\times \vec{hl}\right)/\|\vec{Hl}\times \vec{hl}\|$$

The chirality penalty is then defined with respect to the original value of *p_0_* calculated in the initial geometry. It equals zero if *p∙p_0_ >* 0 (in other words, the sign has not changed, then there is no chirality inversion), but otherwise linearly increases with respect to *|p|*, with a default chirality violation proportionality constant of *k_chir_ = 100*.

The same formalism can be used to force planarity around trigonal substituents. In this case, the *L* substituent is missing, and *p* should ideally equal zero. In the current implementation, no penalty is considered if *|p| < p_oop_ = 0.01*, the latter being an empirically chosen threshold. Otherwise, the penalty increases like *k_oop_(|p| − p_0_)*. At this point, a generic *k_oop_ = 200* is in use for all planar centers, including amide nitrogens.

#### 2.5.4. Potential Energy Surface: Avoiding Singularities

In Molecular Dynamics simulations, singularities of the energy terms in *1/d^n^* at zero inter-atomic are off bounds, because the simulations comply with the energy conservation principle. This is not the case with more aggressive problem space sampling heuristics, such as genetic algorithms. Therefore, in order to avoid systematic checking for zero distances, the non-bonded squares of distances is implicitly augmented by a small increment *d2offset = 0.01*. This has no impact on the precision of these relatively long-range interactions but effectively prevents division by zero errors.

### 2.6. Datasets

#### 2.6.1. Astex/CCDC Clean Subset

Herein, a further subset of the so-called ‘clean’ Astex/CCDC subset [53], containing 191 complexes has been considered (see Supplementary Material) according to several additional filters:

- No covalently bound ligand
- No complexes where the symmetry-related units are mandatory to explain the binding mode (as seems to be the case, among other, for 1ETA or 3CLA—check performed using the “symexpˮ command in Pymol [54]).

#### 2.6.2. Astex Diverse Set

This [55] is a compilation of reliable and diverse complexes for the modeler community. The 85 selected PDB [56] complexes concern targets of agro/pharmaceutical interest. The X-ray complexes possess high resolution, and feature no clashes. A particular attention has been given to the quality of the electron density around the ligand, not including complexes with high nominal resolution but a lack of density for the ligand. Unlike in the previous set, all the ligands are drug-like.

### 2.7. Force Field Parameter Tuning Protocol

The calibration of the additional force-field terms outlined below started from a reasonable initial estimation thereof, obtained by trial and error in previous peptide folding experiments [57,58]. The tunable parameters are:

- *epsilon*: proportionality constant of the distance-dependent relative dielectric constant ε*_r_ = epsilon × d*.
- *repulsive_factor (default 1.0)*: the weight of the an der Waals repulsive term. Therefore, $EvdW_{ij}=\;repulsive\_factor\times A_{ij}/d_{ij}^{12}-\;B_{ij}/d_{ij}^{6}$, where *A_ij_* and *B_ij_* represent the AMBER/GAFF vdW pairwise interaction coefficients.
- *vicinal_weight*: for vicinal (three bonds apart) pairs *i, j*, the entire vdW term *EvdW_ij_* is scaled down by *vicinal_weight*, a trick already employed in the original AMBER.
- *desolv_factor* : the value to be assigned to the desolvation parameter *σ* from Equation (3), for all the pairs *i, j* except the special cases outlined below:
- *minq_to_desolv*: the minimal charge threshold for the desolvation term (*i.e.* if *max(|Q_i_|, |Q_j_|) < minq_to_desolv* then = 0).
- *desolv_scale_ion* and *desolv_scale_hb*: the value of scaling factors for some specific desolvation terms (pairs involving ion or hydrogen-bonds). These extra parameters were required in order to achieve successful docking predictions—a preliminary attempt to fit a set of parameters including only *desolv_factor* to control desolvation failed (results not shown).
- *hbond_bonus*: the common value for all κ*_ij_* in Equation (5) corresponding to hydrogen bond interactions.
- Hydrophobic constants *K_c_* for each hydrophobic carbon class (see Table 1), introduced in order to define hydrophobic contact constants κ*_ij_* in Equation (5) as the average of constants of the involved carbons.

Therefore, three different force field setups can be defined (Table 2). The first, termed ‘Core’ FF, simply represents the default vacuum AMBER/GAFF terms. The second, ‘Preliminary’ FF assumes above-mentioned estimates for the parameters of the additional terms, with a single weight (*K_polarized_ = K_arom_ = K_aliph_* = 0.1) for all carbons types, and no scaling of the desolvation term (*desolv_scale_ion* = *desolv_scale_hb* = 1.0). 

**Table 2:** Considered FF parameter schemes.

Parameter	Core FF	Preliminary FF	Fit FF
epsilon	2	4	4
desolv_factor	0.0	0.1	0.1
minq_to_desolv	0	0.125	0.125
hbond_bonus	0	2	2
repulsive_factor	1.00	0.75	0.75
vicinal_weight	0.5	0.033	0.033
desolv_scale_ion	–	1.0	0.1
desolv_scale_hb	–	1.0	0.1
K_polarized_	0.0	0.1	0.01
K_arom_	0.0	0.1	0.15
K_aliph_	0.0	0.1	0.15

Eventually, the ‘Fit’ FF is the result of a two-stage fine tuning of some of these parameters, as detailed below:

- First, tuning of the desolvation parameters (*desolv_scale_ion* and *desolv_scale_hb*), based on the observations that the Preliminary set-up leads to both (a) bad coordination between ligand and metal ion for several metallo-protein complexes (e.g., mono-dentate coordination preferred to bi-dentate coordination for the acid group in 1CBX), and (b) distorted hydrogen bonds for complexes involving sugars. Thus, the idea to specifically rescale desolvation term of the above-cited classes of interactions by means of weights, fixed by trial-and-error docking simulations of concerned protein-ligand complexes.

**Figure 4:**
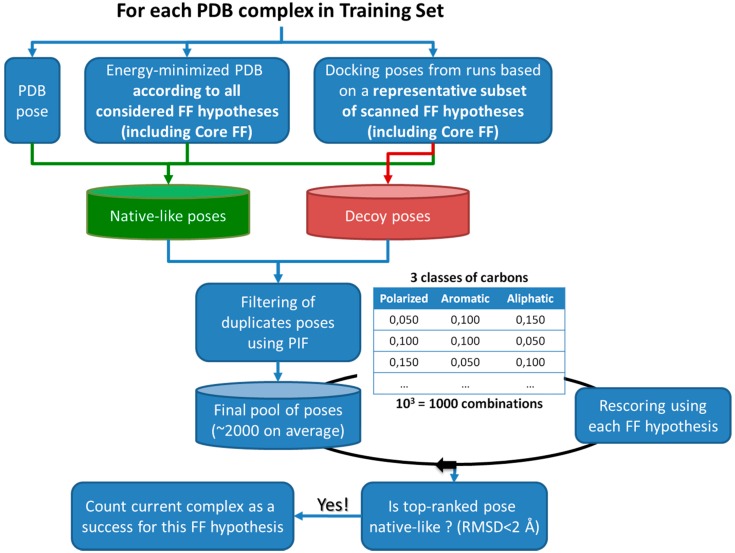
Strategy used in the force field parameter tuning protocol. ‘Decoy poses’ are non-native-like, to be distinguished from the native-like by a proper choice of FF parameters. These three parameters were subjected to a systematic scan. Within the scanned parameter space volume, the representative subset of possible FF configurations includes: Core FF, Preliminary FF setups, setups at the extremes (corners) and at the center of the scanned parameter ‘cube’. This representative subset is used to generate docking poses (one 400-generation run per FF configuration, per complex).
- Last, a systematic scan of hydrophobicity parameters associated to considered classes of carbons was performed. The 33 carbon force field types were regrouped into 3 different hydrophobicity classes: aliphatic, aromatic and polarized, each being attributed a hydrophobicity constant *K_c_* (see Table 1). A total of 10 discrete values (0; 0.010; 0.025; 0.050; 0.075; 0.100; 0.150; 0.200; 0.250; 0.300) are considered as possible *K_c_* choices, allowing for 1000 combinations to scan. The scan was bound to highlight a triplet which systematically ranks, for a large majority of Astex/CCDC-clean complexes, native-like poses as lowest-energy states, out of large and decoy-rich sets of poses. Pose sets were generated by S4MPLE, in iteratively repeated runs employing various FF parameterization schemes (see Figure 4). In practice and for each complex, the pool of poses is rescored using the fitted parameters and one of the 1000 combinations. The top-ranked pose is saved for each complex and all tested combinations, thus it is possible to extract triplets which most frequently favor the expected binding mode. Validation of the herein obtained Fit FF configuration was done by docking of Astex Diverse ligands, and assessment of redocking success.

### 2.8. Redocking Protocol Using S4MPLE

The preparation of the ligands consists of several steps:

- computing partial charges (Gasteiger type) using ChemAxon libraries [59]
- adding GAFF atomic types using the Antechamber tool [39,60]
- using the Parmchk tool [39,41] to check whether there are missing parameters (e.g., bonds, angles or torsions). In that case, Parmchk computes the missing parameters using empirical rules, and these new parameters are added to their respective force field parameters files
- generating a single conformer using ChemAxon libraries (avoiding to start from the expected solution, when the docking accuracy may be artificially enhanced)

Binding sites were prepared using MOE and its Protonate3D protocol [61]. Partial charges and atom types are assigned from the specific AMBER topology file during the initialization of the program. As mentioned before, S4MPLE does not request any formal definition of the binding pocket. Local protein subdomains, including only residues with at least one atom at 10 Å or less with respect to known ligand(s), are used for docking. These are cut out of the PDB structures, after the hydrogen assignment step in MOE. Usual redocking protocols often use a value of 6.5 Å [36,62,63]. Here, larger binding domains are employed, in order to maintain compatibility with the classical FF cut-off magnitudes (9–12 Å). S4MPLE relies on a list of protein atoms to be preferentially used to anchor the ligand, by randomly positioning it such as to establish favorable contacts with these listed hot spots. Hot spots were automatically picked, by detecting the putative hydrophobic contact and hydrogen bonding centers in the close neighborhood of the binding subdomain center (no biased choice of protein groups involved in actually observed contacts). Explicit removal of remote protein moieties is however a potential source of artifacts, given the otherwise unrestricted ligand mobility: this may be pushed out of the site, and led to form favorable fake contacts with—in practice—inaccessible protein atoms. Therefore, ligand poses having their geometric centers at more than 8 Å away from the center of the binding subdomain are systematically discarded in order to facilitate *a posteriori* analysis of results.

Co-factors (prepared/parameterized like ligands, but maintaining their experimental geometry) and ions are included in the binding site, and all waters are removed. During the redocking benchmarks, all binding site atoms are considered as fixed.

**Figure 5:**
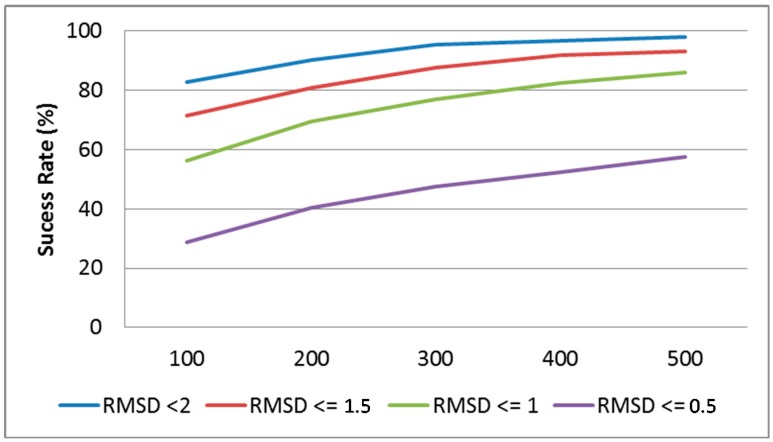
Ability to sample X-ray binding modes (irrespective of their ranking in terms of energy), in function of the number of generations at different RMSD thresholds (Astex Diverse Set).

The redocking simulations consisted in 10-fold runs of Astex Diverse Set complexes. Each run took 500 generations of 50 individuals using the ‘evol’ strategy, mutation probability of 1/10, crossing-over probability 1/10, and *minfpdiff = 0.01.* The number of generations has been set up on the basis of preliminary redocking runs involving both small and large ligands. Sampling success (ability to save a native-like pose using the RMSD metric) has been monitored in function of the number of generations (see Figure 5) and seen to reach a plateau around 400. Thus, the default number of generations has been set up to a slightly larger value (500). After actual docking, so-far visited poses are subjected to a filtering step, involving selection of a non-redundant poses and further optimization (using the exhaustive energy minimization strategy) of the best poses (potential energy within +30 kcal∙mol^−1^ with respect to sampled top conformer).

Success rate (percentage of poses at given RMSD) is then reported as the average of success rates of each independent run. In other words, the 10 independent simulations are monitored independently: if, for a same ligand, six out of 10 succeeded and four failed, six successes and four failures will be counted. The final success average equals to the sum of successes/(10× number of benchmarked complexes).

### 2.9. Redocking Protocol Using FlexX

For direct benchmarking purposes, the program LeadIT (version 2.0.2), developed by BioSolveIT including the docking tool FlexX [12] was used. Protein site files prepared by MOE lead however to unexpectedly bad results in FlexX simulations (results not shown). In particular, the broad definition of the site seemed to prevent FlexX from efficiently discovering native poses, as no docking constraints were imposed. Therefore, a FlexX-specific site preparation protocol was adopted. Hydrogens were now added with LeadIT, but occasional protein protonation states diverging from the MOE predictions used with S4MPLE were manually restored to the latter. FlexX binding sites included all complete residues with at least one atom within a distance of up to 6.5 Å with respect to the reference ligand. Default values for all options relative to ligand preparation were used, except for auto-assignment of protonation states (kept as in S4MPLE runs). Pharmacophoric restraints were disabled for metals. Default FlexX docking options were used.

## 3. Results and Discussion

### 3.1. Force Field Fitting

#### 3.1.1. Tuning of Desolvation Contributions

The Preliminary FF configuration was problematic in modeling of some complexes featuring strong polar interactions, where the Core FF successfully converged toward the expected solution. Bad coordination between ligand and metal ion for several metallo-protein complexes (e.g., mono-dentate coordination preferred to bi-dentate coordination for the acid group of the ligand in 1CBX complex), and low quality hydrogen bonds (e.g., bad D-H.A angle values of ~120° in hydrogen bonds) in complexes involving sugars (1ABE, 1ABF) were examples of such failures. Obviously, the desolvation term at preliminary setup (=0.1) had been too high, cancelling out the benefic electrostatic terms at very low distance (hydrogen-bond case). Besides, dielectric solvent models are notoriously challenged by heavily charged ions, known to cause dielectric saturation phenomena [64]. The two additional scaling constants *desolv_scale_ion* and *desolv_scale_hb*—for pairs including a metal cation and for hydrogen bonding partners (specifically for the polar H/acceptor pair)—were allowed to scan the range from 1 to 0, and a consensus optimum emerged at values of 0.1 (data not shown). Scaling down, by a factor 10, desolvation contributions of metal ions restored the expected bi-dentate coordination in 1CBX. The same holds for hydrogen bonds: scaling down the desolvation term of hydrogen-bond pairs improved predicted geometries. Note that acceptor-acceptor or polar H-polar H desolvation terms are not being scaled down.

#### 3.1.2. Optimizing Hydrophobic Contact Strengths

Over the 1,000 combinations (*K_polarized_, K_arom_*, *K_aliph_*) of hydrophobic factors, the best result at training stage was to obtain 154 correct (top-ranked pose at RMSD < 2.0 Å) out of 191 Astex/CCDC complexes (80.6% success rate). This top result was independently achieved with 11 different combinations. All except one coherently come from a same zone of the combination space: negligible weights for polarized carbons (*K_polarized_* ≈ 0), contrasting with high aromatic and hydrophobic weights (*K_arom_* ≈ *K_aliph_* ≈ 0.15). The outlier (0.2, 0.15, 0.15) is atypical because it considers polarized carbons as the most hydrophobic—also, the 154 complexes being well-scored by it significantly differ from the consensual ones retrieved at all other, physically more meaningful, setups. 

Note that the scanned *K_C_* range properly encompasses the optimal range: at extremes (0, 0, 0) and (0.3, 0.3, 0.3) success rates plummeted to 145 and 142/191 respectively. The absolute worst combination (0.2, 0.1, 0.3) only leads to 138 well-predicted complexes. Eventually, the kept combination in Fit FF was taken at the core of the region: taking the median over listed values for each parameter in Table 3 highlights the selected triplet (0.01, 0.15, 0.15). This is also amongst the best ones with respect to the stricter success criterion RMSD ≤ 1.5 Å (74% success rate). Table 2 lists all parameters and their values in the Fit FF setup.

**Table 3:** Triplets of weights which lead to the best rescoring results (the selected triplet is shown in bold).

Number of Successfully Predicted Complexes	Weights (K)		
	K_polarized_	K_arom_	K_aliph_
154/191	0.000	0.075	0.150
	0.000	0.100	0.150
	0.000	0.150	0.150
	0.000	0.150	0.200
	0.000	0.200	0.200
	0.010	0.075	0.150
	0.010	0.100	0.150
	**0.010**	**0.150**	**0.150**
	0.010	0.200	0.200
	0.025	0.150	0.150
	0.200	0.150	0.150

### 3.2. Redocking-Astex Diverse Set

**Figure 6:**
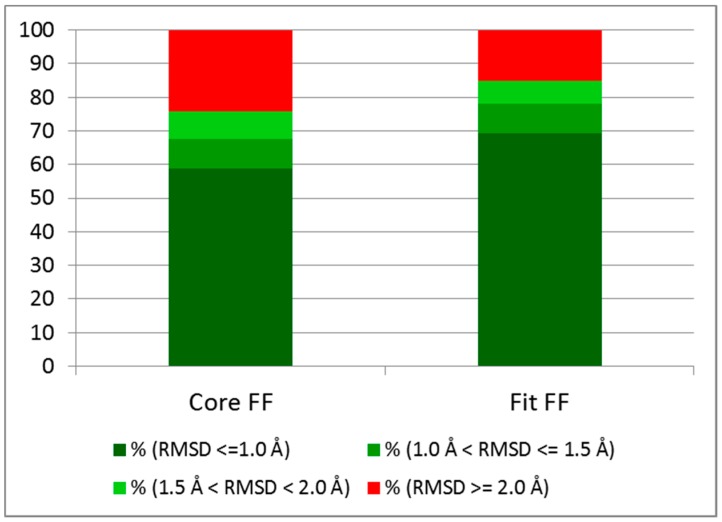
Docking performances on the Astex Diverse Set for both Core FF and Fit FF.

Both Core and Fit FF setups are challenged to dock validation set complexes (Astex Diverse Set [55]). The Fit FF obtained an excellent 85% success rate at RMSD < 2.0 Å for the top ranked pose (average over 10-fold independent runs). The results of redocking simulations are summarized in both Table 4 and Figure 6. This accuracy is equivalent to those of state of art docking tools [34,55,65,66,67]. The lower success rate of RosettaLigand may be due to therein considered protein flexibility.

**Table 4:** Docking performance of several tools on the Astex Diverse Set (* Result from herein described benchmarking calculations, ** Statistics from closest protocols with respect to those presented here). With S4MPLE, Saved poses include the top 30 non-redundant (at *minfpdiff = 0.01*) most stable geometries.

Docking Tools (Scoring)	Success Rate (%) Top Ranked-Pose	Success Rate (%) Saved Poses
S4MPLE (Core FF)	76	93
S4MPLE (Fit FF)	85	96
FlexX *	71	91
GOLD (Goldscore) ** [19]	75–81	Unavailable
Plants (ChemPLP) [65]	87	97
Plants (PLP) [65]	84	
LGA (LargeAll) [34]	63	Unavailable
RosettaLig [66]	58	92
SKATE [67]	87	98

FF tuning clearly matters: there is a significant increase in accuracy of the Fit FF (85%) with respect to the standard vacuum AMBER/GAFF Core (76%) FF. Another important point is the ability to maintain good accuracy at more stringent RMSD thresholds. Thus, for Fit FF, success rate goes down by only 7% at 1.5 Å (78%) and 16% at 1.0 Å (69%) with respect to the usual 2.0 Å threshold. The ability of S4MPLE to almost systematically retrieve a pose close to the experimental one within the top 30 poses is shown too (see Table 4). Global failure cases, and differences between both force field setups are discussed below in more detail. For clarity reasons, only complexes where Core FF and Fit FF-driven docking runs had different success status are shown. In these cases, there is at least one native-like pose (in complexes with systematic errors, the overlay of the native pose and two different ‘wrong’ poses is too crowded to be readable).

#### 3.2.1. Insights on Failure Cases

Three different main scenarios may describe docking failures:

1. All-atom RMSD is high, albeit the top-ranked pose is chemically meaningful, reproducing all the key ligand-site contacts. This may happen:
  (a) if the ligand includes a moiety dangling out in the solvent, or
  (b) the ligand is ‘pseudo-symmetric’, in the sense that it features chemically similar groups which could, in principle, compete for a same binding spot.

In situation (a), the PDB structure of the dangling ligand moiety is likely an over-interpretation of electron density data—or an artifact where crystal packing constrains this otherwise moving moiety. In case (b), say a chlorophenyl-(symmetric linker)-tolyl, the ligand may probably allow for two distinct, comparably populated binding modes (with Cl-Phe and Me-Phe switching binding pockets), whereas electron density fitting would most likely highlight only one of those. Note that S4MPLE fingerprint (PIF) similarity would, unlike RMSD scores, not consider cases 1.a as docking failures. Indeed, calculated fingerprint would be approximately the same as the native fingerprint, whatever happens to the dangling moiety. Scenario 1.b is a real challenge, and can only be evidenced by visual inspection.

2. Neither the top-ranked pose, nor any other of sampled poses, match the native one. This may be due to:
  (a) insufficient sampling,
  (b) inappropriate definition of the binding site (in redocking, this includes ignoring key waters mediating ligand-site interactions), or
  (c) a highly unrealistic force field setup, causing native-like structures to be of high energy, thus effectively preventing them from being sampled and saved.
3. The top-ranked pose differs from the native, but the latter is being sampled and listed among less stable ones. This may be:
  (a) imputable to the force field setup, counting as a failure to identify the expected binding mode as the absolute energy minimum, or
  (b) an inappropriate definition of the binding site (like in 2(b)—however, of more limited impact on results since the expected binding mode has been saved), or
  (c) an entropic effect: minima deeper than the native one exist, as predicted by the FF, but are narrow and therefore not significantly populated at room temperature.

It is not clear at this point how the narrowness of a minimum impacts on its probability to be visited by an EA. According to common sense, hypothesis (c) is unlikely: if such narrow minima exist, their discovery by the algorithm should be an expectedly rare stochastic event. EA sampling cannot furnish positive proof for a 3(c) scenario, so this explanation should be invoked with great caution.

This being said, convergence towards the native structure as so-far lowest energy pose is not an absolute warranty of success—the FF energy landscape may nevertheless feature deeper fake minima, which were not visited, given the limited sampling effort per individual redocking run. However, the native structure is, at least, an attractor in problem space—an important local minimum, if not the absolute one.

There are some general failures to converge towards low-RMSD poses, irrespectively of the FF setup: 1G9V, 1GM8, 1GPK, 1HP0, 1HVY, 1JJE, 1MEH, 1TZ8, 1XM6, 1YGC and 2BR1. These will be detailed below, and assigned to the scenarios mentioned above:

- 1G9V, 1GM8 & 1HVY ligands have limited direct contacts with the binding site: most of the ligand/site hydrogen bonds are bridged with waters. Worse, these ligands feature solvated carboxylate groups, not directly interacting with the target. In 1HVY, the dihydroquinazoline moiety from drug Tomudex is perfectly docked, whereas the two loose carboxylates are, in absence of crystallographic waters, attracted towards neighboring basic residues. Therefore, direct site-ligand contacts are all well predicted—this fits scenario (1(a)). Same holds for 1GM8, the hydrophobic phenylacetamide moiety is correctly positioned, unlike the anionic -lactam moiety. 1G9V, however, is totally dependent on specific water-mediated interactions, therefore classifying as failure 3(b) (the correct pose is being sampled, but not top ranked).
- 2BR1 & 1XM6 are further examples of (3(b)) complexes where water-mediated interactions play an important role. By contrast to the examples above, these water-mediated contacts occur deeply within the binding site. In the top-ranked pose of 2BR1, the ligand is bound head-to-tail, in burying the methoxyphenyl moiety into a deep pocket filled with water in the X-ray structure. In 1XM6, the location of the propoxyphenyl group is perfect but the oxazolidone moiety is erroneously predicted to directly interact with a Zn^2+^ ion (in the experimental structure, a water molecule occupies this area).
- 1GPK & 1MEH are classical FF failure cases (3(a)), native conformations being sampled but not top-ranked. The best poses favor an ionic bond instead of two hydrogen bonds, observed in the PDB file, showing that fine tuning of ionic/polar interactions and desolvation penalty is not perfect. In 1GPK the top-ranked pose is inverted, but nevertheless fulfills expected hydrophobic contacts with the site. In 1MEH, the solvent-exposed carboxylate unsurprisingly prefers the neighborhood of R414, instead of the crystallographic hydrogen bonds to S262. This has a very limited impact on the correctly predicted pose of the buried aromatic moiety.
- 1HP0 is an interesting case, where a non-native conformer with flipped deazaadenine group is nevertheless almost correctly docked—fulfilling all of the experimentally observed contacts. The ligand adopts a correct binding mode around the calcium ion, and reproduces the stacking interactions of the flipped aromatic moiety. Non-bonded energies are similar, thus the preference for the native conformer should have been played out in terms of intra-ligand strain contributions. This is not happening (the native conformer is visited, but ranked slightly less well). At this point, it is difficult to formally rank this as a force field failure 3(a), rather than a genuine multiple binding mode example 1.b. 1JJE is in a similar situation, featuring a pseudo-symmetric ligand, derivative of 2,3-dibenzylsuccinic acid in which one of the Phe groups ports a -O-CH_2_-O- bridge (benzodioxole). The latter makes, however, no additional strong contact to the protein, whereas the central, succinyl moiety coordinating both Zn^2+^ ions is perfectly predicted. It seems that the benzodioxole group may be equally well accommodated on each side of the binding site. Docking does not favor the one expected. It is not clear, however, whether the experimentally observed electron density is witness of a single binding mode, or whether it is a statistical expression of a slight preference among two almost equally populated modes. The head-to-tail binding of this pseudo-symmetric ligand results in an impressive RMSD of 8 Å.
- In 1TZ8, the native PDB pose surprisingly exhibits several slight ligand-site clashes (e.g., distance L17_CD1-Ligand_C = 3.0 Å). Expectedly, even the Fit FF with slightly downscaled repulsive vdW terms, leads to a wrong top-ranked pose—albeit it enumerates the native one. It should be noted that this complex is often reported as a failure [66,67]. Furthermore, authors of the Astex compilation dataset reported this complex as borderline in term of quality of the ligand’s electronic density [55]. Hence, this case should be most likely classified as 3(b) rather than a FF problem (3(a)).
- 1YGC: the most solvent-exposed moiety (arylsulphonamide) adopts a wrong alternative conformation (direct hydrogen-bond with the C58 backbone instead of two water-mediated hydrogen bonds with Y94 and T98), whereas the rest of this large ligand is properly docked.

Most of these cases are often described as common failures in other docking papers [55,66,67]. However, all had the native pose visited by S4MPLE, albeit not top-ranked. Yet, only few amongst the above cases are obviously due to FF errors.

For some other complexes (1KZK, 1MZC, 1UML, 1R58, 1Y6B and 2BM2), the native pose was not systematically visited during each of the 10 runs. However, once found, the native poses turned out to be top-ranked. Since these ligands are large compounds and the best energy over all runs is close to the X-ray binding mode, these failures are the result of a lack of sampling. Hence, these complexes occasionally exemplify the 2(a) scenario: 500 evolutionary generations at population size of 50 may not guarantee convergence (albeit the 10-fold repeated runs are eventually sufficient). This fact is not surprising for heuristics-based algorithms.

#### 3.2.2. FF-Based Scoring: Core *vs.* Fit

As previously described, a real improvement is observed using the Fit FF. There are eight complexes for which the fitted scheme converged towards the expected solution, while the Core FF failed, *vs.* only one exemplifying the opposite situation.

**Figure 7:**
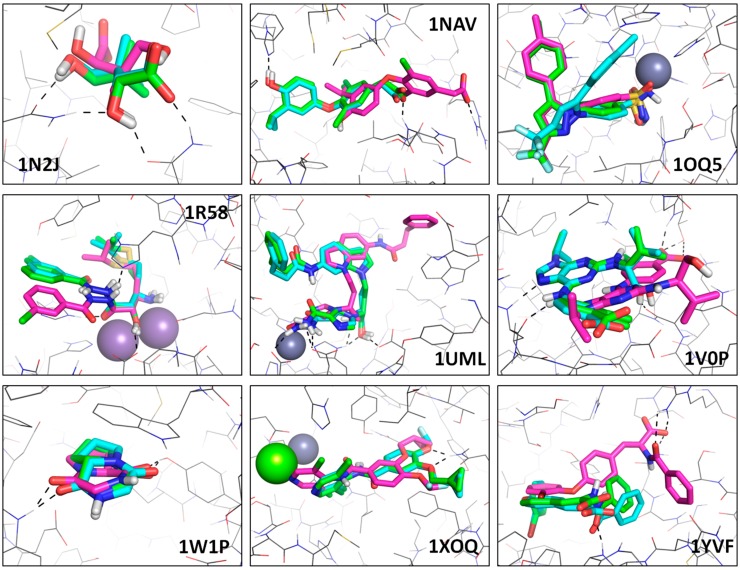
X-ray and top-ranked poses obtained using Fit FF and Core FF from the 9 PDB complexes discussed in the chapter §0 (Core *vs.* Fit). Native poses are shown in green, whereas Fit FF poses and Core FF are displayed in blue and purple respectively. Hydrogen-bonds are shown as black dotted line and ions are represented as spheres.

**Table 5:** RMSD of top-ranked poses for Core FF and Fit FF from complexes discussed in the dedicated chapter (Core *vs.* Fit).

PDB	Core FF	Fit FF
1N2J	3.85	1.42
1NAV	6.26	0.35
1OQ5	0.92	2.83
1R58	3.03	0.74
1UML	7.59	0.66
1V0P	7.60	0.41
1W1P	2.94	0.39
1XOQ	4.04	0.30
1YVF	6.11	0.89

The below mentioned cases (see Figure 7 and Table 5) are, unless otherwise noted, representatives of the 3.a-type failures (native pose sampled, but not ranked as most stable) with Fit FF (1OQ5) or Core FF (all the others):

- 1OQ5: Fit FF has the second-ranked pose matching the expected binding mode, but the most stable one appears clearly non-native whereas Core FF selects the native one. In this Fit FF failure, the ligand is rotated around the pyrazole-benzenesulphonamide axis, with the tolyl group occupying an alternative sub-pocket.
- 1N2J: this small ligand (pantoate) makes four direct hydrogen bonds with the binding site. With Core FF, the top-ranked pose does not make any of these, but places the carboxylate in the hydrophobic area where the two -Me groups are expected. Favorable contact terms (hydrogen-bond and hydrophobic enclosure), and desolvation (penalizing the burial of a -COO^−^ in a hydrophobic pocket) in Fit FF successfully restore the correct binding mode at the top of the list.
- 1NAV: the compound is buried and makes several hydrogen bonds with the binding site. Fit FF finds the native binding mode with a great precision (RMSD ≈ 0.5 Å), whereas Core FF selects a translated pose (several Å away from the experimental binding mode: RMSD ≈ 6 Å) in order to create an ionic bond between the carboxylic group of the ligand and the solvent-exposed R266. This is another example of overestimated polar contributions in absence of scaled down electrostatics and desolvation.
- 1R58: in that case, both scoring schemes lead to an acceptable solution for buried and metal coordination groups of the ligand, but the chlorophenyl moiety is wrongly docked by Core FF, which has no particular incentive to flip this group in order to bring in within hydrophobic contact range with Y444, as seen in the experimental structure.
- 1UML: this ligand is located near a Zn^2+^ ion but does not directly interact with it in the X-ray structure. Fit FF leads to a perfect pose, while the Core FF completely misplaces the ligand, in order to allow for an interaction with that cation.
- 1V0P: Fit FF returns a perfect top-ranked solution, in which the carboxylate group of the ligand is solvent-exposed and doesn’t make any direct contact with the protein. At the opposite, the Core FF favors a pose where the ligand is less buried, allowing the carboxylate group to make several fake hydrogen bonds with the binding site.
- 1W1P: this complex contains a small cyclic dipeptide (Gly-L/Pro). Although both hydrogen bonds with the site are conserved using the Core FF, the RMSD is high because the ligand is flipped with respect to the experimental binding mode. This is rather a 1.a scenario, in which ligand flipping does not affect observed interaction patterns. Conversely, the Fit FF top-ranked pose matches the exact binding mode.
- 1XOQ: Although two cations are present in the binding site (Zn^2+^ and Mg^2+^), the drug (roflumilast) does not directly interact with them in the crystal structure. The Core FF forces a binding mode where the dichloropyridine group of the ligand is close to the magnesium ion, whereas the Fit FF returns the expected binding mode with two hydrogen-bonds with the same sidechain (Q369).
- 1YVF: this case is similar to 1V0P, featuring a solvent-exposed carboxylate, forced by Core FF into several non-native interactions with R394. The best pose from the Fit FF reproduces almost exactly the experimental binding mode. Generally speaking, Core FF favors less buried ligand poses, with fake polar contacts: it overestimates Coulomb terms, ignores desolvation and does not reward hydrophobic contacts.

## 4. Conclusions

S4MPLE is a general conformational sampling tool, based on evolutionary algorithms. It was designed to support full atom flexibility, and based on a set of powerful general generic operators, including an original conformer population diversity control mechanism (differentiable pairwise interaction fingerprints). As a consequence, S4MPLE sees no fundamental difference between actual sampling of a single molecule, or docking of one—or several—ligand(s) into a target site. This is due to the fact that its genetic operators automatically detect the context in which they are called—recombination of covalently bound or non-bonded fragments—and seamlessly adapt to it. Potential degrees of freedom are automatically detected, but can be controlled by the user: fixing of molecular moieties allows the approach to concentrate on relevant DoF, be they intra- or intermolecular. Thus, the theoretical applicability range of S4MPLE is broad: from small peptide folding and ligand sampling, to rigid site docking, to classical flexible docking (with moving side chains), to extreme flexible docking (allowing larger movements of protein loops, backbone included), to multiple ligands docking. Therefore, the fact that the herein reported “classical” benchmark tests showed that S4MPLE is neither better nor worse than state-of-the-art software is good news—at equal classical docking performances, S4MPLE is better in terms of its much larger applicability domain.

Care was taken to adapt the genetic operators to the specific nature of the energy landscape. Herein used ‘driven’ mutations or fragment recombinations are much more likely to produce relevant geometries, by contrast to the typical random tinkering with torsional angle values, bound to lead to clash-rich, impossible geometries.

Pairwise monitoring of the status of all putative contacts (including, unlike in classical IF [44,46], intra-ligand and—if site flexibility is enabled—intra-site terms) fuzzily represents the molecular geometry of the whole system. PIFs are not meant to extract similar binding modes shared by chemically different compounds, albeit this information can be easily mined for, by analyzing the populated PIF elements.

S4MPLE can be employed, in response to the complexity of the considered problem, either as stand-alone single CPU process, or as computer-grid deployed, massively parallel simulation (work in progress).

It was shown that outfitting the AMBER/GAFF force field with very simple desolvation and hydrophobic contact terms clearly improved rigid-receptor redocking success scores. At a strict count of redocking successes, at a customarily employed criterion of RMSD < 2 Å, within the widely used Astex Diverse data set, S4MPLE ranked better or similar to state-of-the-art docking tools. Furthermore, many of the cases counting as failures were shown to be situations in which multiple ligand binding modes cannot be easily excluded (e.g., almost symmetric ligands). Furthermore, all the non-native top-ranked poses correspond (if a sufficient number of generations is allowed) to deeper energy wells compared to the level reached when relaxing the native structure in our enhanced AMBER/GAFF force field. These failures, when not attributable to ignored water-mediated interactions due to deletion of intervening crystallographic waters, outline (expectable) limitations of the FF-based potential energy model. In no instance S4MPLE systematically failed to reach the baseline energy level of the relaxed native structures, showing that its sampling procedures are powerful, successfully avoiding entrapment in local optima, notably using regularly approaches avoiding consanguinity within the population.

At the moment, S4MPLE is still a prototype under development. The runs were quite time-consuming (several hours/single CPU for the average rigid-site redocking simulation), but:

(a) a plethora of technical but important parameters—such as, for example, stopping criteria of local search procedures, *etc.*—were not yet optimized, being set by default to rather strict values.
(b) the main goal behind this development is not to add one more rigid-site docking program to an already long list. So far, the purpose of this development was to assess in how far experimental docking poses can be correctly predicted on the basis of the herein defined energy function (no re-ranking based on fitted free energy scoring functions) if unbiased sampling is allowed.

The use of S4MPLE for classical rigid-site docking could be easily enhanced—for example, by a manual, knowledge-based selection of site hot spots, by contrast to the automatic approach used here. Also, the availability of smooth, differentiable interaction fingerprints allows for straightforward inclusion of problem-specific knowledge (imposing specific contacts to occur on the protein side). However, having passed these first classical tests, further work will address problems of larger complexity—fragment-like compounds docking, multi-ligand docking and binding site flexibility.

## Supplementary Material

The following:

- x86_64 executable of S4MPLE,
- User guide,
- various ligand preparation tools and
- force field parameter distributions
- spreadsheet with the list of the training set compounds used to calibrate Fit FF, and detailed docking results for each of the repeated docking runs are available for download from: http://infochim.u-strasbg.fr, DOWNLOADS section (tar.gz). Docked poses are available upon request (mol2 format) (http://creativecommons.org/licenses/by/4.0/).
